# Playful Antisedentary Interactions for Online Meeting Scenarios: A Research Through Design Approach

**DOI:** 10.2196/62778

**Published:** 2025-04-18

**Authors:** Jiaqi Jiang, Shanghao Li, Xian Li, Yingxin Xu, Jian Zhao, Pengcheng An

**Affiliations:** 1 School of Design Southern University of Science and Technology Shenzhen, Guangdong China; 2 Department of Computer Science University of Illinois Chicago Chicago, IL United States; 3 School of Computer Science University of Waterloo Waterloo, ON Canada

**Keywords:** gamification, sedentary behavior, videoconferencing, exertion games, embodied interaction, design research

## Abstract

**Background:**

Online meetings have become an integral part of daily life for many people. However, prolonged periods of sitting still in front of screens can lead to significant, long-term health risks. While previous studies have explored various interventions to address sedentary lifestyles, few have specifically focused on mitigating sedentary behavior during online meetings. Furthermore, design opportunities to address this issue in the context of online meetings remain underexplored.

**Objective:**

This study aims to investigate the design of effective antisedentary interactions for online meeting scenarios and understand user experiences with gamified bodily interactions as an antisedentary measure during online meetings.

**Methods:**

This study adopts a “research through design” approach to develop and explore user experiences of gamified bodily interactions as interventions to mitigate sedentary behavior during online meetings. In collaboration with 11 users, we co-designed and iterated 3 prototypes, which led to the development of the Bodily Interaction Gamification towards Anti-sedentary Online Meeting Environments (BIG-AOME) framework. Using these prototypes, we conducted user studies with 3 groups totaling 15 participants. During co-design and evaluation, all group semistructured interviews were transcribed into written format and analyzed using a conventional qualitative content analysis method.

**Results:**

The findings demonstrate that gamified bodily interactions encourage users to engage in physical movement while reducing the awkwardness of doing so during online meetings. Seamless integration with meeting software and the inclusion of long-term reward mechanisms can further contribute to sustained use. In addition, such games can serve as online icebreakers or playful tools for decision-making. Drawing from 3 design prototypes, this study offers a comprehensive analysis of each design dimension within the BIG-AOME framework: bodily engagement, attention, bodily interplay, timeliness, and virtual and physical environments.

**Conclusions:**

Our research findings indicate that antisedentary bodily interactions designed for online meetings have the potential to mitigate sedentary behaviors while enhancing social connections. Furthermore, the BIG-AOME framework that we propose explores the design space for antisedentary physical interactions in the context of online meetings, detailing pertinent design choices and considerations.

## Introduction

### Background

Online meetings have become a significant aspect of modern work, introducing new daily health challenges. An increasing number of activities are being conducted primarily or entirely online [1]. As noted by Wu and Yu [2], individuals tend to prefer online meeting platforms due to their advantages such as flexibility and cost-effectiveness, even when working from home is not mandatory. However, increasing research evidence has revealed the presence of technostress in virtual conferencing, which can negatively impact mental and physical well-being, productivity, job satisfaction, and group commitment [1,3-5]. Among these challenges, prolonged sedentary behavior, with its potential for causing irreversible health damage over time, stands out as a significant threat [6-8].

Sedentary behavior occur in periods of wakefulness during which the body engages in minimal movement, such as sitting, lying, or reclining [9]. The negative effects of prolonged sedentary behavior—distinct from merely a lack of physical activity—cannot be fully mitigated by intermittent physical exercise [10]. According to current medical studies, no “gold standard” exists for measuring sedentary behavior, nor are there specific guidelines on how to effectively interrupt such behaviors [11]. The prevailing recommendation is to reduce excessive sedentary behavior by replacing it with mild to vigorous physical activity [12,13]. Seated activities that incorporate moderate movements of the lower or upper body can help reduce the negative effects of sedentary behavior [12]. This approach underscores the necessity to integrate more active routines in daily life to counteract the health risks associated with prolonged sedentary periods.

Many interventions have been developed to reduce sedentary time and encourage more active engagement in physical activities [14,15]. These include break reminder software for computers [16,17], mobile apps designed to interrupt prolonged sedentary behavior [18], dedicated devices equipped with LED displays [19], posture-adjusting smart chairs [20], and indoor location–based mobile games [21]. However, online meeting environments, as distinct sources of technostress due to remote social interactions, remain underexplored in design research. Therefore, there is a pressing need for research to collect a broader range of design examples and provide specific guidance for developing effective and engaging interventions to address sedentary behavior in online meetings.

In-person meetings typically feature habitual breaks, encouraged by social and environmental elements [22]. However, online meetings often lack these cues or social expectations, leading to fewer affordances for breaking sedentary routines [23]. This indicates the need for designing antisedentary activities that could fit into online meetings and engage users in a socially amusing way. Analysis of large-scale video meeting data shows that multitasking is a common behavior in online meetings. While multitasking can lead to negative outcomes, such as increased distraction [24], it may also have positive effects, such as improved efficiency in attention division or task shifting [25]. This highlights a design opportunity to harness the positive aspects of multitasking in online meetings to reduce sedentary behavior by incorporating physical exercises into the meeting process as secondary or parallel activities. Gamified bodily interactions offer a promising approach to integrating simple, brief physical exercises into meeting routines.

### Gamification and Exertion Games

Gamification is defined as “the use of game elements in non-game contexts” [26]. It applies the principles of game design and elements such as storytelling, leaderboards, and winning rules to address real-world challenges in areas such as training, health care, and education [27]. Previous studies have demonstrated that gamification can facilitate behavior change [28-30] and make products more engaging [31]. Exertion games or exergames [32], which promote physical activity by incorporating exercise into digital games, have become an increasingly relevant area of research. According to Mueller et al [33], all computer systems that facilitate physical exertion as part of the interaction could be regarded as exertion games, regardless of whether their primary focus is on gaming or exercise.

Many studies have focused on motivating physical activity through gamification by developing software or using hardware [34-39]. Exertion games have demonstrated not only their effectiveness in promoting physical activity but also their ability to enhance social interaction among users [40,41]. Mandryk et al [42] analyzed exertion games from the lens of mitigation of sedentary behaviors and articulated 2 design principles: “providing an easy entry into play” and “motivating repeated play sessions throughout the day.” Gamified bodily interactions have been applied in areas such as fitness, education, and training [30,43,44]. However, to the best of our knowledge, few studies have investigated how gamified bodily interactions can be integrated into videoconferencing scenarios to effectively promote physical activity and combat sedentary behavior.

At the intersection of remote education and exertion games, Shin et al [45] proposed Jumple, a virtual physical education classroom that leveraged artificial intelligence pose estimation technology to facilitate physical activity for students in online environments. By leveraging commonly available devices, Jumple aimed to address the challenges of remote learning and the negative impact of the COVID-19 pandemic on children’s well-being. Sachan and Peiris [46] investigated the use of augmented reality–based micro–health interventions to mitigate “Zoom fatigue”—a term describing the somatic and cognitive exhaustion caused by the intensive or inappropriate use of videoconferencing tools [5]—among college students during virtual classes, exploring their effectiveness in reducing sedentary behavior and promoting physical activity. These studies offer design instances from online classroom settings, which is a similar context to online meetings. Building on this foundation, our exploration pertinently focuses on the online meeting context and aims to generate further knowledge through a design framework to systematically reveal the design space and implications.

### Understanding the Online Meeting Environment

For decades, scholars have predicted that online meetings will reform the conventional routine of commuting to and from the workplace and the way people collaborate with others [47]. Nevertheless, for many people, the heightened reliance on online meetings has proven to be mentally and physically challenging. Rudnicka et al [48] found that break taking is a behavior mediated by the social norms among coworkers, while remote work environments often lead to extended periods of inactivity and excessive work due to the absence of social cues. Given the drawbacks and challenges associated with current videoconferencing software, we propose to add socially engaging bodily interactions to afford more antisedentary components in online meetings. Many people perceive breaks involving sedentary screen activities (eg, playing games, using social media, browsing the web, and watching videos) as unhelpful, while considering physical activities to be helpful breaks [49]. However, in practice, people tend to exceed the intended duration for digital and static breaks, whereas physical breaks, especially outdoor activities, are less likely to extend beyond the planned time [50]. To address this, we explore the potential of adding low-threshold physical movements as a playful antisedentary option. As a supplement to higher-threshold physical activities (eg, standing up or walking around), these games provide an additional way to promote helpful breaks during online meetings.

Multitasking is a common phenomenon during meetings. In face-to-face meetings, people engage in various physical activities such as pacing, standing, and stretching, which are beneficial to overall meeting performance [25]. During telephone calls, people also tend to perform minor movements while talking without disrupting the communication [47]. However, online meetings include more of digital multitasking than physical multitasking (eg, locomotion or other movements). On a Zoom call, maintaining a central position within the camera view with one’s face clearly visible to others is regarded by cultural norms as professional and trustworthy [51]. Research often stresses the negative impacts of multitasking, including increased mental workload and reduced productivity, which could potentially contribute to burnout and depression [5]. However, according to a large-scale analysis of remote meeting multitasking behaviors, in-meeting multitasking can also lead to positive outcomes when participants are able to regulate their attention, making flexible use of time during less demanding or noncritical parts of a meeting [25]. The authors suggest we should allow “space for positive multitasking” and “shorten meeting duration and insert breaks” [25]. In the future, people’s tendency to multitask is unlikely to diminish due to the growing prevalence of digital devices and the nature of the modern workplace [52]. Inspired by the idea of leveraging positive multitasking to mitigate sedentary routines, our design incorporates bodily game elements into the online meeting interface.

### Objectives

Given the limited knowledge on *how to design gamified bodily interactions as antisedentary interventions in online meetings*, our study adopted a research through design methodology [53] to address 2 research questions (RQs):

- How can gamified bodily interactions be integrated into online meetings to reduce sedentary behavior?
- What are the relevant design options and considerations to properly design such gamified bodily interactions for online meeting contexts?

To address the first part of this research, we conducted 3 rounds of co-design activities with 11 participants. During these sessions, participants brainstormed, elaborated, and assessed initial bodily game ideas and low-fidelity prototypes considering their prior online meeting experiences. On the basis of the co-design insights and outcomes, we propose the initial Bodily Interaction Gamification towards Anti-sedentary Online Meeting Environments (BIG-AOME) framework that describes a design space to help designers and researchers consciously navigate multiple relevant design dimensions. Across these dimensions, the BIG-AOME framework reveals various design options that can be considered to make thoughtful design choices according to specific design goals and scenarios.

To further consolidate and contextualize the BIG-AOME framework, in the second part of this research, we implemented 3 game design ideas into functioning prototypes and evaluated them with 15 participants in online meeting settings. The 3 designs were chosen because they were hypothesized to represent distinct design options across various dimensions, enabling a concrete exploration of the design space and uncovering context-specific insights and implications underlying the BIG-AOME framework. These bodily game prototypes can be integrated into mainstream online meeting platforms (eg, Zoom or VooV Meeting) via virtual camera software such as OBS Studio (Open Broadcaster Software) [54]. The visual components of the games are overlaid on the camera view or video tiles of online meeting attendees. While the games are active, the attendees can interact with each other and the game objects via bodily movements adapted from existing beneficial physical exercises. This approach allows gamified exercises to be embedded flexibly into meeting sessions as a playful way to mitigate sedentary meeting routines, with the participants still being seamlessly engaged in the meeting and connected with other attendees.

Our study offers a design-oriented exploration of gamified bodily interactions as a novel approach to breaking sedentary online meeting routines. Our contributions are two-fold: (1) a total of 3 prototypes as design instances of antisedentary bodily gamification for online meetings, along with empirical findings regarding user experiences; and (2) a preliminary design framework for creating gamified bodily interactions for online meetings, highlighting a promising design space with relevant design options and considerations to inform and inspire future research.

## Methods

### Design Explorations and the Formulation of the BIG-AOME Framework

In this section, we present our iterative research through design approach [53], including our early design explorations, co-design workshops, and the initial formulation of the BIG-AOME design framework.

#### Early Design Explorations

##### Overview

In the initial stage, we explored the possibility of integrating gamified bodily interactions as antisedentary interventions in online meeting scenarios. To do so, we first studied existing literature [26-30] and online resources (eg, credible health information websites such as MedlinePlus [55], the Centers for Disease Control and Prevention [56], and the World Health Organization [57], as well as YouTube channels maintained by professional physical therapists or exercise coaches). The purpose was to accumulate suitable antisedentary physical exercises as design inputs. These exercises specifically target areas such as the shoulders, neck, and back and can be performed in typical online meeting settings. This corpus of verified healthy movements served as an inspiration resource for gamification design ideas to build upon. Subsequently, we generated a wide range of gamification ideas, implemented them in functional low-fidelity prototypes, and tested them for firsthand experience and as preparatory materials for later co-design workshops.

##### Insights From Early Design Explorations

The most important lesson we learned was that simply using bodily interactions to control a “classic” digital game would often fail to provide appropriate gaming experiences. Two unsuccessful early attempts—arm-controlled versions of Angry Birds and Snake—were perceived as blunt and tedious, with arm gestures leading to fatigue rather than alleviating the strain of sedentary behavior. As Mueller et al [58] noted, these designs treated the body as merely a controller for digital objects, failing to leverage the advantages of “body as play.” Therefore, we focused on creating gameplay experiences that could naturally integrate the players into online meeting environments to achieve in-depth and seamless bodily engagement.

The early design explorations laid the groundwork for our BIG-AOME framework. In the initial stages, we developed design considerations based on literature research, online resources, and insights from early prototyping. These considerations addressed various aspects and formed the initial elements of our framework. In the subsequent co-design sessions, we refined these considerations based on users’ needs and experiences. This process led us to the final version of the framework.

#### Co-Design Workshops

##### Overview

To address user needs in real-world contexts and refine the design space, we used a participatory design approach, inviting individuals with diverse online meeting experiences to contribute to the development of gamified bodily interactions. We organized 3 online co-design workshops to elicit participants’ meeting experiences, create new design ideas and functional low-fidelity prototypes, and gather design insights. We recruited 11 participants (n=3, 27% male; n=8, 73% female) with diverse educational and professional backgrounds, including business, design, and engineering. Of these 11 participants, 7 (64%) were aged 18 to 24 years, 2 (18%) were aged 25 to 34 years, and 2 (18%) were aged 45 to 54 years. We refer to these participants as P1, P2, and so on up to P11. Each workshop session lasted approximately 2 hours. To facilitate the co-design process, we prepared prompt cards to inspire the participants during their discussions and idea sketches. These cards were designed based on our literature study and firsthand experience. The participants were presented with 8 card sets, reflecting our initial design considerations as mentioned previously. Each set addressed a key consideration, including start time, duration, bodily movements as input, bodily interplay, action, game elements, attention, and exercise intensity; for example, the card set titled Action Cards included 21 illustrations, with each depicting a beneficial bodily exercise found in the literature and credible online sources. Similarly, the Attention Card set featured a spectrum ranging from games that allow participants to play while focusing on the meeting to those requiring full concentration.

**Figure 1:**
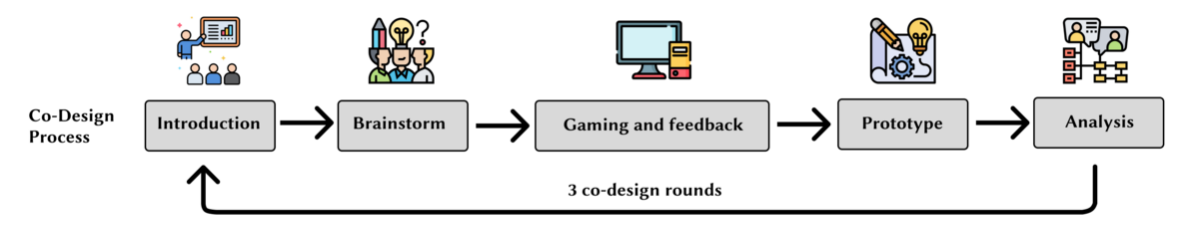
An illustration of our design process, highlighting the various stages involved.

The workshops were hosted on Zoom (Zoom Video Communications, Inc) to simulate an online meeting setting and allow the participants to test functional low-fidelity prototypes within the Zoom interface. Design activities were carried out using Figma (Figma, Inc) and its FigJam feature. As illustrated in Figure 1, the co-design workshop consisted of 5 steps: introduction, brainstorming, gaming and feedback, prototyping, and iterating. Participants were briefed on the research background, purpose, and workshop schedule in the introduction stage. During brainstorming, they shared their ideas, provided suggestions, and generated new concepts. The gaming and feedback stage involved playing gamification low-fidelity prototypes or acting out design ideas together and exchanging opinions in a group discussion. In the prototyping stage, participants sketched out their ideas based on insights from the previous stages. Finally, they refined their low-fidelity prototypes and shared their design considerations with others. After the workshops, we acquired a rich understanding of underlying real-world needs from the participants. We collected 2 types of data during this process. First, we amassed a significant number of ideas from the brainstorming stage of the co-design workshops. Second, we documented group discussion records. To further clarify the insights from the idea cards, we used an affinity diagram to identify common themes, patterns, and relationships among the data points. For the discussion content, we used the conventional qualitative content analysis method outlined by Hsieh and Shannon [59] to examine how the initial design considerations supported participants in formulating their ideas.

##### Insights From Co-Design Workshops

The co-design workshops yielded rich insights. We identified 4 major themes in the affinity diagram (Textbox 1). First, the social aspect was identified as critical, with competition and cooperation influencing user engagement. Second, participants expressed a preference for games with low learning curves, which aligned with a design principle proposed by Mandryk et al [42]: “providing an easy entry into play.” The third point concerned the physical fatigue and mental stress associated with online meetings, highlighting the need to differentiate between the intensity of physical activity and the attentional demands of games. Finally, we observed variations in the layout view and display order of video tiles among users, with tile sizes varying based on the number of meeting participants. Fluctuations in the number of attendees, speakers, and layout settings within the meeting platform can impact the meeting interfaces. These factors should be considered when designing games.

**User needs identified from workshops and representative responses from participants.**

**Consideration of social factors**

- “When I see the name of one of my best friends on it, my desire to win is very strong.” [P8]
- “I am more inclined toward something that can be played alone.” [P6]

**Desire for simplicity and familiarity**

- “Everyone is already familiar with the rules of these games [a reference to games such as Fruit Ninja and Whack-a-Mole, which were the inspirations behind game ideas suggested by participants].” [P3]

**Adaptability to physical and mental states**

- “The mind is already very tired [during the meeting].” [P11]
- “If [the intensity of physical activity is] too gentle, I can’t feel my body being relaxed.” [P2]

**Clear visibility and usability**

- “I can’t clearly see the sticker elements on the video.” [P9]

The discussion content analysis revealed that the design consideration cards we supplied played a substantial role in shaping and refining participants’ ideas. The analysis identified 3 key ways the cards were used: inspiring and guiding nonexperts in proposing designs, adapting designs to the online meeting context, and evaluating design choices. These findings also offer insights into the application of the proposed BIG-AOME framework.

#### BIG-AOME Framework

##### Overview

**Figure 2:**
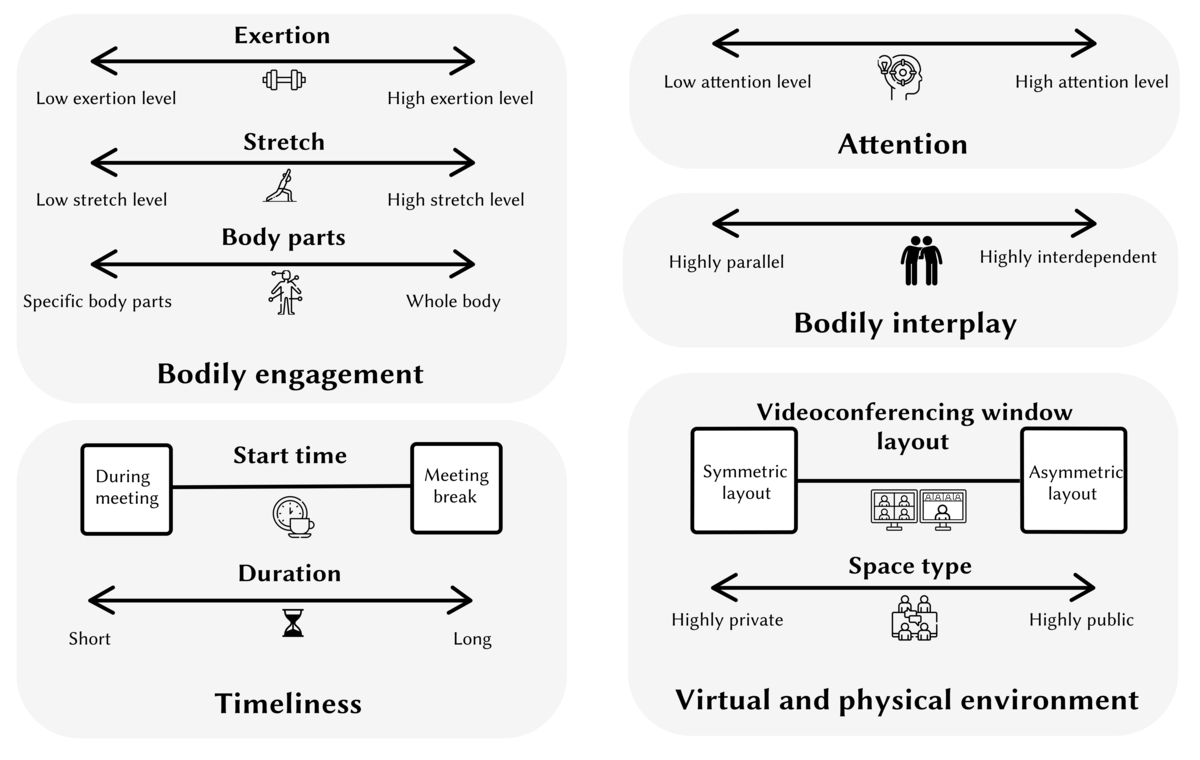
The Bodily Interaction Gamification towards Anti-sedentary Online Meeting Environments framework.

In this section, we present the BIG-AOME framework. Initially formulated during early design explorations, the framework has been enriched and refined based on insights and design implications derived from the co-design sessions. As illustrated in Figure 2, the framework consists of 5 dimensions that specify a broad design space for creating gamified antisedentary bodily interactions for online meeting scenarios. Here, we briefly introduce each dimension, with a more concrete examination provided later using empirical data from the user evaluation.

##### Bodily Engagement

The *bodily engagement* dimension describes players’ physical experience during gameplay. It includes 3 subdimensions: *exertion*, *stretch*, and *body parts*. *Exertion* represents the perceived intensity of the physical effort exerted during the game. *Stretch* refers to the perceived extent of physical reach and flexibility required by the game. The *body parts* subdimension indicates the perceived extent to which various parts of the body are engaged during gameplay: some games primarily involve a specific movement, such as head nodding, while others require full-body activities, such as dancing. Incorporating the 3 subdimensions allows us to design games that are more pertinent to the nuanced needs and design goals.

##### Attention

The *attention* dimension measures the level of concentration needed for gameplay and highlights positive multitasking during online meetings. Games requiring peripheral attention enable users to engage in movement without losing focus on the meeting. Conversely, games demanding greater focus provide high engagement and offer attendees a pause, promoting physical activity and fostering interactive play among participants. With numerous design nuances, the *attention* dimension spans a broad spectrum for game design, ranging from lightly engaging to fully immersive experiences.

##### Bodily Interplay

The *bodily interplay* dimension builds upon the concept proposed by Mueller et al [41]. It evaluates to what extent users’ bodies interact with and influence each other during the gaming experience. Highly interdependent bodily interplay requires collective participation, fostering a shared gaming experience. By contrast, highly parallel bodily interplay allows participants to engage with the game independently, without requiring cooperation from others. This form of interaction is noninterfering and can be asynchronous. The level of bodily interplay is a vital design consideration, depending on the design objectives and the social dynamics of the meeting attendees. Therefore, it requires thoughtful deliberation in the design process.

##### Timeliness

The *timeliness* dimension comprises 2 subdimensions: *start time* and *duration*. The *start time* subdimension determines the most suitable moment to commence the game. Some games are designed to be engaged with during the meeting, whereas others might be better suited to ad hoc breaks. The *duration* subdimension focuses on the length of an episode of the gaming experience. Episodes can be brief, offering a quick gamified experience that ends once completed, or continual, allowing intermittent engagement over a longer period. The design choices for both subdimensions depend on the meeting context and attendees’ preferences, requiring careful consideration.

##### Virtual and Physical Environments

This dimension addresses 2 crucial factors: the layout of the videoconferencing window and the nature of the user’s physical space. Videoconferencing software typically provides either symmetrical (eg, thumbnail video tiles) or asymmetrical (eg, speaker view) layouts. In an asymmetrical layout, typically used when a participant shares their screen, the shared content occupies a larger portion of the display. This setup enables the shared screen to display gameplay elements to all participants, collectively drawing their attention to the game. The larger display area in this layout may accommodate more visual elements. Conversely, in a symmetrical layout, the screen is divided equally among participants. This layout allows users to see more faces but may result in smaller videoconferencing windows, making it difficult to discern finer visual details, particularly in large meetings. Designers should recognize the differences between symmetrical and asymmetrical layouts and consider the nature of the meeting. Turning to the physical environment, the *space type* pertains to the level of privacy and ownership of a user’s surroundings. A highly private space, such as a personal study, enables users to move and speak freely without social concerns. Conversely, a highly public space, such as a library or an office, may limit movement and vocal interactions to avoid disturbing others or creating social awkwardness. These constraints can affect users’ willingness and ability to engage in games during meetings.

### Three Design Prototypes

**Figure 3:**
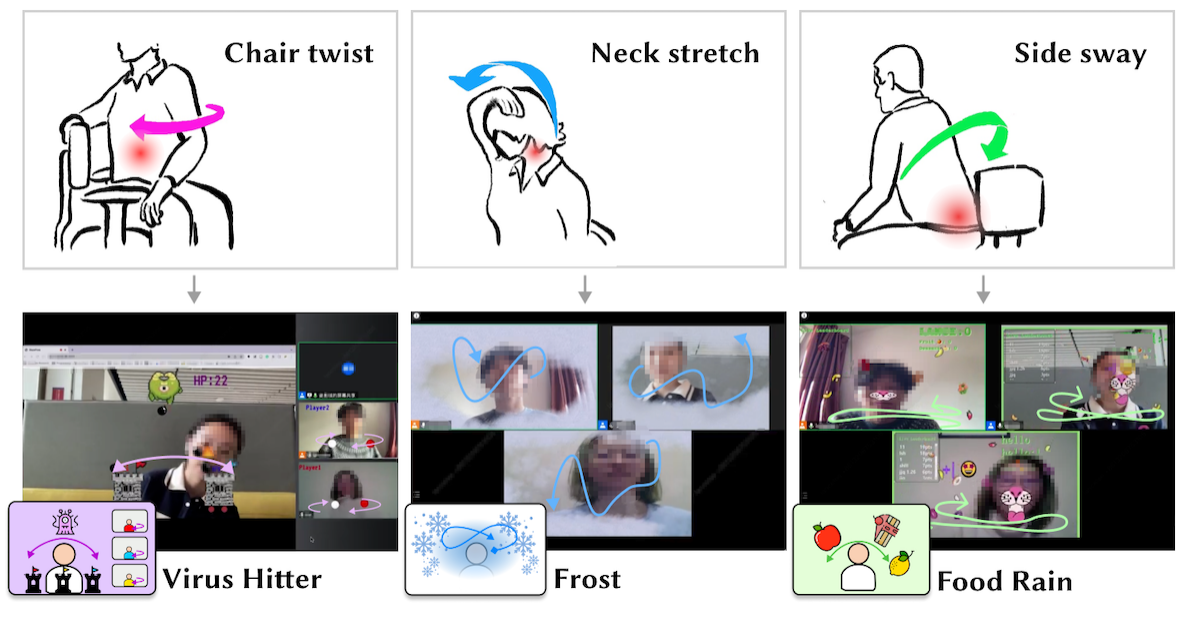
The 3 high-fidelity prototypes developed to probe the design of antisedentary bodily gamification for online meetings.

To explore how gamified bodily interactions can be integrated into online meetings to reduce sedentary behavior (RQ1) and to contextually examine and refine the BIG-AOME framework for relevant design implications (RQ2), we developed 3 high-fidelity prototypes for user evaluation: Virus Hitter, Frost, and Food Rain (Figure 3 [60,61]; Multimedia Appendix 1).

Virus Hitter is a multiplayer cooperative game in which players are assigned roles: 1 participant is randomly selected as the “hitter,” while the others serve as the hitter’s assistants. The assistants’ interaction is adapted from an existing exercise called “chair twist” [60]: participants, while seated, are asked to rotate their upper bodies side to side, facilitating a relaxing effect. The hitter, meanwhile, moves a torch that appears on the screen and is attached to their nose via motion tracking, using it to launch bombs at watchtowers. The hitter’s interaction is derived from an exercise called “side sway” [61], which involves side-to-side sway while remaining seated. Every assistant corresponds to 1 watchtower matched by color. A brief animated tutorial is offered so that players can quickly and easily understand how to play the game. Virus Hitter is designed to promote a high level of bodily interactivity and requires the cooperation of all participants, thereby not only encouraging physical activity but also fostering social bonding among players.

Frost is a gamified interaction designed for use during online meetings. It simulates frost slowly forming on the surface of the videoconferencing window, gradually spreading from the edges toward the user’s image. To prevent their personal window from becoming entirely obscured by frost, users must move any part of their body to “swipe” it away. The suggested movement for this activity is inspired by the“neck stretch” [60], which involves the simple action of raising and lowering the head to stretch the neck muscles. This specific interaction was chosen to provide a natural and inconspicuous reason for meeting participants to engage in light physical activity without feeling self-conscious. This game is characterized by low levels of exertion, stretch, and attention, making it ideal for relieving muscle and spine fatigue through gentle and unobtrusive movements. These movements do not significantly distract from meeting discussions or disrupt the flow of the meeting, whether in virtual or physical settings. Frost is designed to be played seamlessly as a secondary task, complementing the primary activities of an online meeting, thus enabling participants to stay active without interrupting the meeting dynamics.

Food Rain is a multiplayer competitive game. After inputting a nickname, players see fruits and desserts falling from the top of the screen. They move their bodies and open their mouths to catch the falling food items in their own window to score points. Catching a fruit increases the score by 1 point, while catching a dessert decreases it by 1 point. This movement is also derived from the side sway exercise [61]. During this activity, players’ neck and waist muscles receive a moderate workout. As some users might feel awkward opening their mouths in front of others, the design superimposes a cartoonish animal mouth that tracks users’ mouth movements (opening and closing) in real time. A leaderboard in the top left corner of each user’s window displays the rankings and scores of all players. The competition mechanism encourages long-term participation. Food Rain, which has a moderate duration, was designed with the assumption that it would trigger relatively high social engagement.

The 3 gamified bodily interactions are implemented on a web-based application and streamed via OBS Studio [54] as a virtual camera, which enables its integration with any online meeting platform (eg, Zoom and VooV Meeting). As online meeting platforms and their application ecosystems continue to evolve, gamified bodily interactions have the potential to emerge in various forms in the future. They could be available as add-on applications within the platforms, integrated as features of the meeting interface itself, or even implemented as virtual backdrops for meetings.

**Figure 4:**
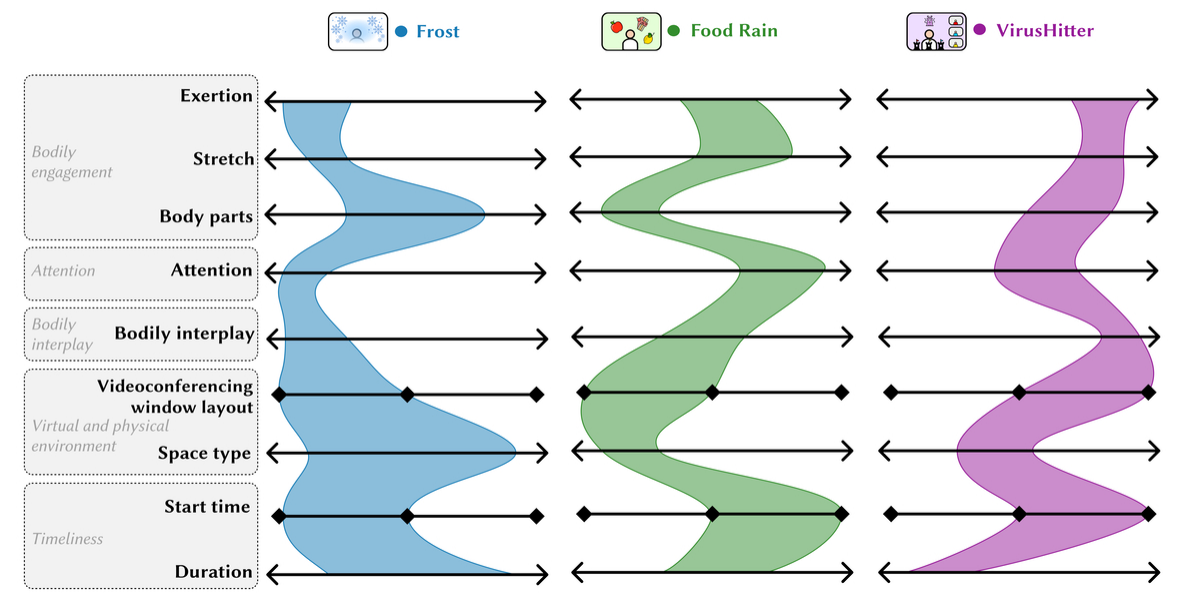
Distribution of dimensions on the framework when designing the 3 prototypes.

The prototypes presented here are representative examples chosen to illustrate and explore the design space. They were selected from a range of game designs developed during our early exploration and co-design phases. In the process of transforming ideas into prototypes, we considered various dimensions of the framework. As depicted in Figure 4, each prototype is intentionally situated in different regions within these dimensions. This strategic placement results in each prototype embodying unique design patterns. Each dimension contributes to the overall game design, and different combinations of dimensions can yield a diverse range of games.

### Evaluation Study

#### Participants and Setup

**Figure 5:**
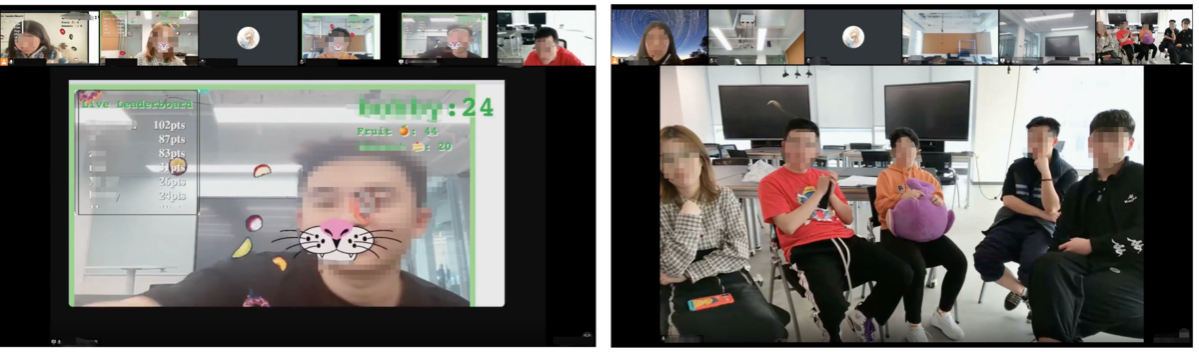
Screenshots showcasing: the user evaluation process (Left) and the focus group interview process (Right) conducted on the online meeting platform.

The study involved 3 rounds of sessions with 4, 5, and 6 participants, totaling 15 participants (n=10, 67% male; n=5, 33% female), referred to as P12, P13, and so on up to P26. Of these 15 participants, 11 (73%) were aged 18 to 24 years and 4 (27%) were aged 25 to 34 years. A prestudy survey was conducted to gather participants’ basic information and online meeting habits. The results showed that 53% (8/15) of the participants attended ≥1 online meetings weekly, and 87% (13/15) spent >1 hour in each meeting. To simulate a realistic online meeting scenario, we organized 3 rounds of online seminars focused on ChatGPT. Each session included discussions and integrated gamified activities. The seminars were designed as collaborative learning experiences, where participants were introduced to foundational ChatGPT concepts through lecture videos and group discussions featuring open-ended questions. Fixed-interval breaks were incorporated into the meetings, during which participants engaged with gamified exercises. After the seminar, participants independently evaluated the games by completing an evaluation panel designed using Figma. This panel, based on the BIG-AOME framework, aimed to capture their feedback on the gaming experience and design elements (refer to Multimedia Appendix 2 for details). Each session concluded with a focus group interview, where participants discussed their gaming experiences and shared insights into their evaluation choices (refer to Multimedia Appendix 3 for details). To ensure a smooth process, all games were preconfigured on participants’ browsers, and clear guidelines were provided for the evaluation and interview stages. The entire meeting process, including the focus group discussion, was recorded for subsequent analysis. As shown in Figure 5, screenshots illustrate the user evaluation process and interview process conducted on the online meeting platform.

#### Procedure

**Figure 6:**
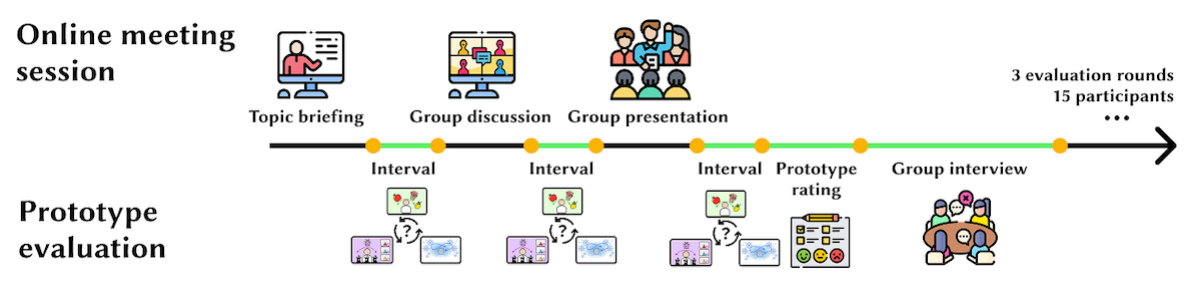
A visualization of the overall evaluation procedure.

As illustrated in Figure 6, the entire study spanned 105 minutes, comprising 45 minutes for learning activities, 30 minutes for gameplay, 10 minutes for completing the evaluation panel, and 20 minutes for a focus group interview. Each game was allocated 10 minutes, providing participants ample time to engage as they wished.

The study began with the host introducing its objectives and process, after which participants watched a YouTube video providing an overview of ChatGPT and its underlying mechanism. They then took their first meeting break to play a randomly selected game. After the break, participants were divided into groups to discuss the risks and opportunities associated with using ChatGPT. Each group was assigned a team member to facilitate the discussion. Another game was then randomly selected for all participants to experience. After the gameplay, 1 representative from each group presented their opinions to the rest of the participants. The final game was played during the last meeting break.

After completing the seminar simulation, participants were invited to evaluate the games and share their personal insights on how these games could be applied in real-life scenarios. Participants were given 10 minutes to complete the evaluation panel. We emphasized that they should provide responses based on their own experience and that all perspectives were valid. Finally, a 20-minute focus group interview was conducted to explore participants’ experiences with gamified bodily interactions and discuss potential application scenarios. The group discussions followed a semistructured format, using open-ended questions to facilitate open and comprehensive conversations. First, we asked participants to recall their gaming experience, including physical sensations, mental responses, and interactions with others. Next, participants explained the reasons behind their ratings on the evaluation panel. Finally, we invited participants to describe how they would use the framework to create a similar game if given the opportunity.

#### Data Collection and Analysis

**Figure 7:**
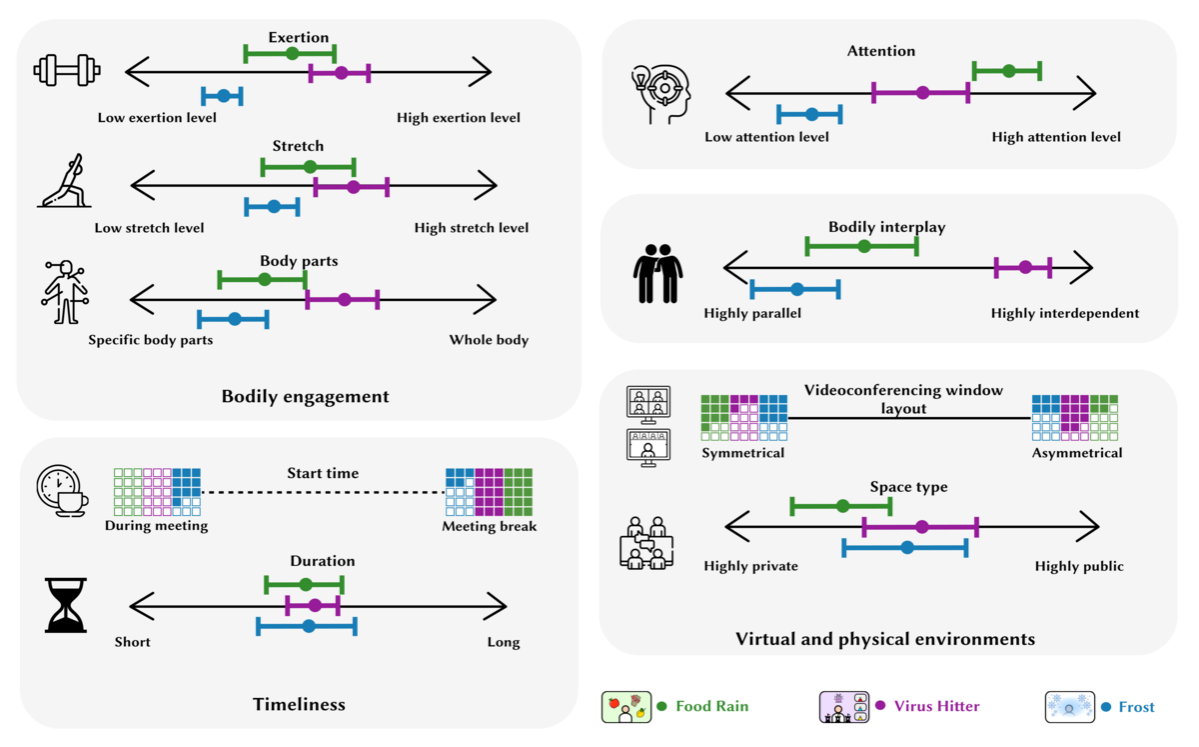
Results of users’ evaluation regarding the distribution of the 3 games on the framework. The horizontal line for each game represents the IQR (Q1-Q3), defining the middle 50% of the data, which highlights the central distribution and concentration of the data values.

Data collection and analysis comprised 2 parts. First, we conducted focus group interviews to gather users’ experiences with gamified bodily interactions during online meetings. All interview data were transcribed verbatim. We used the conventional qualitative content analysis method outlined by Hsieh and Shannon [59] for an in-depth understanding of participants’ perceptions of and experiences with the games played during meeting breaks. This process led to the development of preliminary codes, which were iteratively refined and adjusted as our understanding of the data evolved. These codes were then organized into clusters, providing a clear outline of the major findings from our qualitative data. Second, participants rated each of the 3 games based on their experiences within the dimensions of the BIG-AOME framework. These ratings were instrumental in identifying the framework regions occupied by each prototype and in validating our initial design assumptions. The ratings were processed using Python (Python Software Foundation) and are descriptively visualized in Figure 7.

### Ethical Considerations

This study has been approved by the Medical Ethics Committee of Southern University of Science and Technology (approval number: 20230044; application number: 2023PES044).

Informed consent was obtained from all participants before their involvement. The informed consent form provided a detailed explanation of the study's procedures, potential risks, and expected benefits. It also outlined the confidentiality of participants’ personal data and emphasized that all ethical standards would be upheld throughout the research. Participants were made aware that their involvement was entirely voluntary, and they could withdraw from the study at any time without facing any penalties. To ensure privacy protection, all collected data were anonymized and securely stored in an online central database. Each participant received compensation of US $15.

## Results

### Integrating Gamified Bodily Interactions Into Online Meetings (RQ1)

The first aim of our study was to explore how gamified bodily interactions can be effectively integrated into online meeting settings (RQ1). This investigation seeks to provide empirical insights into this novel antisedentary approach, identifying potential opportunities and addressing challenges to inform future applications. Our findings highlight 3 key themes (Textbox 2): (1) perceived value in breaking sedentary behavior patterns, (2) potential factors influencing motivation and sustained use, and (3) anticipated use scenarios and additional benefits.

**Key themes and descriptions.**

**Perceived value in breaking sedentary behavior patterns**

- Provides a reason to move
- Reduces the awkwardness of moving during meetings

**Potential factors influencing motivation and sustained use**

- Seamless integration with meeting software
- Long-term reward mechanisms

**Anticipated use scenarios and additional benefits**

- Online icebreaker activities
- Gamified decision-making

#### Perceived Value in Breaking Sedentary Behavior Patterns: “Now I Have a Reason to Move”

Participants affirmed that the designed bodily interactions provided a compelling reason to move during online meetings, offering innovative ways to disrupt sedentary routines. On the basis of their prior experiences, they could all relate to the physical strain and “discomfort” (P21) caused by the sedentary nature of online meetings. Introducing bodily interactions, as P22 noted, could effectively reduce sedentary behavior and encourage physical movement in online meeting scenarios by bringing “more physical activity in online meetings.” A participant noted that when they were in an online meeting and wanted to move, they felt “embarrassed” (P15). Another participant shared as follows:

This game gives me a chance, now I have a reason to move.

P22

This view was reinforced by a participant:

You often remain static during online meetings, but adding such games will add more subjective movement.

P23

In addition, P12 and P14 indicated that after playing the games they felt more relaxed: “a little more relaxed than at the beginning for sure” (P12. Overall, participants expressed that gamified bodily interactions not only gave them a reason to engage in physical activity but also reduced the awkwardness associated with moving during online meetings.

#### Potential Factors Influencing Motivation and Sustained Use: “This Will Motivate Me to Stick With It”

##### Overview

Participants agreed that the games were effective in encouraging movement because they were novel, easy to learn, and facilitated highly interactive sessions that reduced social awkwardness. However, they acknowledged that the novelty and social benefits might diminish over time, particularly in routine online meetings where attendees are already familiar with the games. To address this, participants emphasized the need for strategies to sustain engagement over the long term. This supports our view that gamified bodily interactions should be integrated into meeting routines, rather than being transient or overly intense, promoting lasting behavioral change. The interviews revealed 2 key factors influencing user experience that are critical for achieving sustained success.

##### Seamless Integration With Meeting Software

A participant suggested adding prompts:

I think a pop-up prompt might remind me to use it more.

P19

Accordingly, it might be beneficial to incorporate reminder prompts on meeting platforms during breaks or between long sessions. However, the timing and delivery of these reminders need to be carefully considered. There is existing research indicating that notifications delivered at inappropriate times can distract users and potentially annoy them [19]. P21 expressed that seeing others engaging in antisedentary interactions could motivate them to participate as well. With seamless integration, users could be effectively prompted to initiate the bodily interactions.

##### Long-Term Reward Mechanisms

Extrinsic motivation arises from external factors, such as rankings and individual records [62]. Implementing these motivators (eg, points, badges, and leaderboards) in gamified bodily interactions can foster a competitive environment, thereby encouraging participants to remain engaged and enhance their performance. A participant noted that the leaderboard in Food Rain heightened competitiveness:

We played pretty aggressively because of the leaderboard.

P13

Another participant described the influence of the leaderboard as akin to “peer pressure” (P16). Furthermore, offering a variety of games can cater to diverse participant preferences and prevent monotony. Regular updates to existing games, including introducing “new game patterns” (P19) and “new visual and aural effects” (P13), are both convenient and effective strategies for sustaining player involvement. Indeed, this encapsulates the rationale behind our research use of gamification: to enhance extrinsic motivations for physical activity and reduce sedentary behavior.

#### Anticipated Use Scenarios and Additional Benefits: “It Could Be Used as an Icebreaker Activity”

When discussing the potential applications of gamified bodily interactions in online meetings, participants highlighted the possibility of integrating these games into various scenarios. Gamified bodily interactions can serve as icebreakers at the beginning of online meetings or during breaks, helping participants to become acquainted with each other and creating a comfortable atmosphere, as well as helping to break down communication barriers, especially in situations where participants have not met before or have had limited prior interactions; for instance, P13 expressed appreciation for the game Virus Hitter as a potential group icebreaker. P17 and P24 also highlighted the potential of such interactive games for serving as effective icebreakers in virtual settings:

It could be used as an icebreaker activity.

P24

This insight underscores the versatile applicability of gamified bodily interactions in online meetings, extending beyond mere physical engagement to fostering social connections.

In online meetings where decision-making is required, games can be used to facilitate discussions, promote active participation, and encourage team members to share their opinions. The interactive nature of these games can help maintain engagement during lengthy discussions and ensure that all voices are heard; for instance, a participant envisions the integration of these games as a creative tool for participant selection while simultaneously energizing the meeting atmosphere: “for example, to decide on a speaking order” (P13). In situations where a group of participants need to choose a member to speak or present but are unsure about whom to select, they can initiate the game and obtain a ranked list of candidates. Motivated by the ranking system and the prospect of being chosen to speak, participants are likely to engage actively in the game and incorporate physical activity into their meeting experience. This innovative approach to participant selection not only encourages physical movement but also adds an element of excitement and competition, fostering a livelier and engaging online meeting environment.

### Contextualizing the BIG-AOME Framework (RQ2)

#### Overview

In this section, we contextually examine and concretize the framework with the empirical data gathered from the evaluation sessions. As illustrated in Figure 7, participants were asked to position the 3 evaluated prototypes on each subdimension to verify initial design assumptions and gain an overview of how these design instances are distributed over this design space. Descriptive statistics are presented in Table 1. Using the gathered data, we analyze each design dimension and elaborate on relevant design choices to highlight the design space and considerations for future research.

**Table 1:** Descriptive statistics of user rating results across games and dimensions.

Dimensions and games		Ratings, mean (SD)	Ratings, median (IQR)
**Exertion**			
	Food Rain	0.462 (0.251)	0.413 (0.306-0.528)
	Virus Hitter	0.551 (0.156)	0.598 (0.466-0.649)
	Frost	0.207 (0.109)	0.210 (0.158-0.263)
**Stretch**			
	Food Rain	0.474 (0.276)	0.500 (0.278-0.636)
	Virus Hitter	0.609 (0.210)	0.663 (0.453-0.750)
	Frost	0.349 (0.144)	0.304 (0.250-0.418)
**Body parts**			
	Food Rain	0.278 (0.172)	0.302 (0.157-0.430)
	Virus Hitter	0.570 (0.212)	0.569 (0.419-0.750)
	Frost	0.206 (0.220)	0.143 (0.090-0.236)
**Attention**			
	Food Rain	0.781 (0.256)	0.781 (0.703-0.984)
	Virus Hitter	0.523 (0.276)	0.577 (0.422-0.690)
	Frost	0.228 (0.160)	0.235 (0.141-0.306)
**Bodily interplay**			
	Food Rain	0.414 (0.270)	0.454 (0.220-0.594)
	Virus Hitter	0.858 (0.176)	0.952 (0.799-1.000)
	Frost	0.178 (0.273)	0.067 (0.000-0.248)
**Duration**			
	Food Rain	0.478 (0.271)	0.485 (0.337-0.601)
	Virus Hitter	0.491 (0.167)	0.500 (0.390-0.573)
	Frost	0.503 (0.270)	0.523 (0.282-0.669)
**Space type**			
	Food Rain	0.322 (0.310)	0.250 (0.080-0.395)
	Virus Hitter	0.570 (0.311)	0.500 (0.330-0.784)
	Frost	0.458 (0.340)	0.336 (0.225-0.722)

#### Bodily Engagement: How Does the Game Involve Bodies and Challenge Physically?

The first dimension of the framework focuses on the physical involvement and challenges presented by the game. This dimension encompasses 3 subdimensions: *exertion*, *stretch*, and *body parts*.

As shown in Figure 7, we can observe that the 3 prototypes follow a similar pattern in the 3 subdimensions: Virus Hitter involves the body more than Food Rain, which in turn involves the body more than Frost. This is in line with our initial design intentions. However, it is important to note that the ordering of the games in the 3 subdimensions is not always consistent; for instance, a game involving multiple body parts can still require low exertion, such as matching the body’s key points to a static shape—an idea proposed by the co-design participants.

Designs with lower bodily engagement levels appeal to a wider range of participants and involve less exertion, enabling players to engage more frequently or for longer periods. However, these designs may not effectively engage individuals who prefer high-energy activities or competitive environments, potentially leaving them feeling unchallenged or uninterested. By contrast, designs with higher bodily engagement levels offer more intense physical challenges. However, games requiring high bodily engagement may not be suitable for all participants due to physical limitations or personal preferences, leading some individuals to feel excluded or frustrated if they cannot fully engage in the activities; for example, after playing Virus Hitter, a participant said they were “too tired” (P19). Another participant stated as follows:

The last game is more obvious [a reference to the bodily engagement levels of Virus Hitter], the first two [Frost and Food Rain] are relatively easy.

P12

This highlights the varying levels of bodily engagement across the 3 games, confirming the noticeable differences in the physical challenges they offer.

#### Attention: What Level of Focus Is Required?

It can be observed from Figure 7 that Food Rain demands the highest level of attention, followed by Virus Hitter and Frost. The 3 games span almost the entire *attention* dimension, providing players with varying levels of cognitive engagement. This diverse range of attention levels can cater to the different preferences and scenarios in online meetings.

Bodily interactions that require low attention levels allow participants to engage without disrupting the meeting. They can maintain focus on the meeting content or personal tasks simultaneously, ensuring that the overall flow of the meeting remains uninterrupted while participating in peripheral bodily interactions. In addition, incorporating a design that requires only peripheral attention during a meeting break can be an effective way to encourage relaxation and mental rejuvenation. Participants can engage in the game while also performing other tasks, such as drinking water:

I can do something else [during a break while experiencing the gamified interaction].

P19

However, games that require only peripheral attention might not fully engage participants or provide a deeply immersive experience. P21 shared that Frost is “not like a game” and therefore they felt less enthusiasm to play it. Games that require high attention levels can create a more engaging and immersive experience. Moreover, such games can offer a complete shift from the meeting content, allowing participants to refresh their minds before returning to the meeting activities. However, playing games requiring high attention levels can lead to greater mental stress or cognitive load for participants.

#### Bodily Interplay: To What Extent Can Participants’ Bodies Act Upon and React to Each Other in Gamified Bodily Interactions in Online Meetings?

As shown in Figure 7, Virus Hitter demonstrates a significantly higher level of bodily engagement than Food Rain and Frost. Collectively, the prototypes cover a wide range of bodily movements in this dimension.

Games requiring highly parallel bodily interplay provide flexibility in terms of participants’ availability, allowing individuals to engage or disengage without impacting others. This feature is especially suitable for online meeting scenarios where participants may already be under mental strain. With minimal reliance on other players, individuals may feel less pressured to excel, fostering a more relaxed, enjoyable experience for those seeking less competitive interactions. However, such games may become repetitive and less captivating over time due to the limited participant interaction and challenge levels. Conversely, games requiring highly interdependent bodily interplay can foster collaboration and communication, potentially strengthening team dynamics. Such games, particularly when they incorporate competitive or cooperative elements, can enhance participants’ engagement and motivation. Nonetheless, they also present challenges because the players’ enjoyment and success depend heavily on their teammates’ or opponents’ actions, which can impact the overall experience.

#### Timeliness: When Should the Interaction Begin? How Long Should It Last?

As illustrated in Figure 7, regarding the preferred start time for each game, all participants suggested that both Food Rain and Virus Hitter are better suited for initiation during session breaks. Most participants felt that it would be more appropriate to initiate Frost in the middle of meeting activities. These insights correspond well with our design expectations, suggesting that our strategy regarding optimal game timings aligns with participant preferences.

Incorporating a game during a meeting break presents several benefits, including minimizing meeting disruption, facilitating social interaction among participants, and potentially enhancing team relationships. However, there are also drawbacks. Participants might prefer to use breaks for other tasks, such as attending to personal needs or engaging in work-related discussions. Furthermore, if some participants do not wish to participate in games during breaks, they might feel excluded or pressured to join. Conversely, initiating a game midway through a meeting offers a unique chance to invigorate participants. However, maintaining the right balance of attention is essential. Games incorporated into a meeting must be thoughtfully designed to avoid disrupting the flow or causing participants to lose focus on the main agenda.

Concerning the *duration* dimension, we noticed an intriguing pattern. All 3 games are positioned in the middle of the dimension, displaying minor differences. Our results indicate that user experiences across the 3 prototypes do not show considerable variations within the *duration* dimension. Short-duration games offer ease of integration into online meetings, serving as refreshing breaks that do not demand much time. They serve as effective transitions between meeting segments or as energizing preludes to more serious discussions. Offering frequent opportunities for physical and mental breaks, these games counteract sedentary behavior effectively. Long-duration games promise a more immersive experience with complex mechanics, deeper narratives, and enhanced team-building opportunities. However, fitting them into the meeting agenda without disrupting the flow can be challenging, and extended play might lead to participant fatigue if the game is too demanding.

#### Virtual and Physical Environments: What Is the Optimum Videoconferencing Window Layout? Should Space Type Be Highly Private or Highly Public?

As shown in Figure 7, most participants believed that Virus Hitter should be experienced in an asymmetrical layout, while Food Rain and Frost should be experienced in a symmetrical layout. This aligns with our design considerations.

An asymmetrical layout, where a participant shares their screen, provides an expanded display space, potentially enhancing the game’s visual impact. However, this might inadvertently shift focus toward the sharer’s content, potentially reducing interaction with other attendees or causing discomfort for the sharer under scrutiny. A symmetrical layout, with equally sized videoconferencing windows for all participants, creates an equitable and harmonious environment, encouraging shared focus on the sharer and others. However, the smaller videoconferencing window size may impact game visibility and engagement, especially in larger meetings. Therefore, careful consideration and balance in the design process are essential to optimize user experience and participation.

Concerning the *space type* dimension, Food Rain was viewed as more suitable for private spaces, while Virus Hitter and Frost inhabit the middle ground. Playing in a private space offers comfort and security, allowing for uninhibited movement and gameplay without fear of social awkwardness or disturbing others. However, this may reduce social interaction and potentially lower motivation due to a lack of group dynamics. By contrast, playing in a public space enhances social interaction and participation, fostering a sense of community and group camaraderie. The visible engagement of others can encourage individual participation. However, playing in a public space can lead to feelings of self-consciousness during gameplay, potentially affecting enjoyment and immersion.

## Discussion

### Design Implications and Opportunities for Creating Antisedentary Online Meeting Environments

#### Overview

Our study aligns with the studies by Warburton et al [63] and Aldenaini et al [15], which have demonstrated the benefits of gamification and persuasive technology for antisedentary designs in general. In the context of online teaching, Shin et al [45] and Sachan and Peiris [46] conducted pilot studies to combine bodily interactions with online classroom scenarios. We further explored the vast design space and highlighted its underlying design implications. Furthermore, contributing to a design-oriented approach to this domain, we created prototypes for evaluation, enabling us to gather real-world experiences of participants within an online meeting environment. Here, we discuss 4 key design implications that emerged from our study.

#### Providing Proper and Playful Reasons for Moving the Body During Online Meetings

Sedentary behavior is regulated by personal habit strength [64] and is often socially and environmentally reinforced [13]. Many new technological interventions have been designed to reduce sedentary time, including games, reminders, wearable devices, and smart office equipment [42,65-67]. These interventions have typically adopted action planning as the key mechanism. However, an online meeting is a unique context where users are under additional stressors such as close-up, “face-to-face” communication and reduced mobility [47] or distractions at home and online [24]. Participants also reported that during online meetings they would feel highly embarrassed to stand up or move their body as they wanted even if they were feeling very tired. In such a situation, compared to a time-based prompt for a break, an incentive cue and a compelling reason for a break might appeal more to meeting participants.

Medical evidence shows that the quantified threshold for sedentary time varies by health condition, age, sex, and so on [13]. There are no rigorous recommendations about the optimum limit for sedentary time [68]. A study investigating the motivational processes underlying sedentary behaviors shows that action planning has a conditional effect on physical activity but no effect on limiting sedentary behavior [64]. This suggests that the focus should perhaps not be on setting strict activity goals or tracking exercise sessions. Instead, encouraging movement and breaking up sedentary patterns whenever possible may be more beneficial. This does not necessitate high-intensity training or strenuous workouts. Even light or moderate movements incorporated into online meetings can make a significant difference [69]. From a design research perspective, the challenge lies in creating socially engaging designs that provide meeting attendees with enjoyable, playful, and socially motivating reasons to engage in movement. Rather than strictly monitoring and regulating activity, the goal is to create an environment that naturally encourages movement and reduces sedentary behavior.

#### Designing Unobtrusive Bodily Movements as Secondary Tasks to Leverage Positive Multitasking in Online Meetings

Multitasking is a common behavior in online meetings, and it can have both positive and negative impacts [25]. Incorporating nondistracting, low-effort physical movements as secondary tasks during online meetings may serve as an effective design strategy for positive multitasking. Games with varying attention requirements offer diverse options for attention management, enabling participants to fluidly transition between relaxation and meeting focus; for instance, games that demand minimal attention can serve as a peripheral activity during the meeting. One such example is *Frost*, which features a minimalistic visual interface and is intentionally nondistracting, making it suitable as a secondary task. The level of required attention is a crucial consideration in game design. By consciously defining the design goals and leveraging the benefits of positive multitasking, we can foster an antisedentary environment within online meeting routines.

#### Aiming for Fine-Grained Integration With Routines, Rather Than Intensity or Volume of Physical Activity

Our work presents a unique proposition that deviates from conventional exertion game design aimed at sports training or physical activity tracking. In addressing sedentary behaviors during online meetings, our emphasis is not on the intensity or volume of physical activity. Instead, we prioritize designing interactions that are mild in intensity, of moderate duration easily initiated, and accessible to most participants. These interactions allow for flexibility, enabling users to engage with or dismiss them as needed.

In terms of mitigating sedentary behaviors, as prior research argues, the goal is fulfilled not merely by increasing exertion intensity but also by incorporating light physical exercises [70] into daily working scenarios. It is important to note that sedentary behavior is completely different from a lack of physical activity. Most current exertion games are considered augmented sport [33], relying on game consoles and sensor equipment to create a game-like sports experience, thereby promoting physical activity [36]. Our work represents a different approach to exertion games: our games are designed specifically as playful antisedentary interventions. Instead of using these games solely for workouts or ad hoc entertainment, our objective is to seamlessly integrate them into online meeting routines. This integration aims to foster more lightweight, healthy behaviors among participants during their regular online engagements [42,71].

#### Expanding Design Variety and Integrating Games Into Existing Meeting Platforms to Enhance Real-World Impacts

In our exploration of the potential of gamified physical interactions within online meeting scenarios, we introduce the BIG-AOME framework. This framework serves as an initial foray into a 5D design space, revealing a broad spectrum of design opportunities that can guide the creation of a diverse array of experiences tailored to various user preferences. Our objective with the BIG-AOME framework is to equip both researchers and designers with a foundational tool to further investigate this innovative approach. By promoting the integration of exertion games [33] with persuasive interventions [14], we aim to inspire a collaborative effort in the development of engaging and health-promoting solutions tailored for online environments.

Recognizing the diversity in user preferences and motivational factors, it is vital to adopt a personalized approach to game-based health interventions. An individual’s engagement with the game is influenced by factors such as motivation, interests, athletic abilities, social connections, and social status [72]. Hence, to inspire long-term positive behavior changes, it is crucial to craft inclusive and engaging experiences that resonate with the varied expectations of diverse users. During the evaluation phase, we noticed a diversity of user preferences for the different games, which signals a degree of success in catering to our audience’s varied expectations. Nevertheless, we acknowledge that numerous areas across the various dimensions and subdimensions remain unexplored. This underscores the need for more diversified, design-driven research. By generating new design instances and extracting new design implications, designs in this domain could evolve toward more personalized, engaging, and effective game-based interventions.

Moreover, the importance of seamless integration with existing meeting platforms cannot be overstated. This echoes the principle proposed by Mandryk et al [42]: “providing an easy entry into play.” Given the social nature of online meetings, users are often motivated to engage when they observe other users doing so on the platform. This affords new ways to increase user involvement [41]: with the growing ubiquity of online meetings, these platforms naturally present opportunities for an increased presence of gamified bodily interactions. By incorporating these interactions into existing meeting software and delivering appropriate signifiers about this new feature, users could become more conscious of their sedentary habits and are consequently more likely to participate in physical activities.

### Limitations and Future Work

While our study contributes valuable insights into gamified physical activities within online meetings, it is essential to acknowledge certain limitations that necessitate further research. First, the evaluation period of our study was relatively brief, which may not adequately capture the long-term effects of these interventions. Future research should include longer evaluation periods to investigate more thoroughly the sustainability and long-term impact of such gamified interventions. Second, although we developed 3 high-fidelity prototypes that varied across different dimensions, we did not comprehensively explore some subdimensions. Consequently, while the data collected offers significant insights, these limitations may restrict the generalizability and robustness of our findings. Despite these constraints, we are optimistic about the extensive and promising potential of gamified bodily interactions in online meetings. Future studies could aim to experiment with more diverse and inclusive design features covering a wider range of physical activities. Expanding the scope of the interventions to include a broader variety of bodily movements and postures will enhance our understanding of the potential and effectiveness of integrating gamified physical activities into online meeting environments. In addition, investigating how gamified bodily interactions can stimulate users’ intrinsic motivation to support sustained engagement over the long term is another valuable direction for future research.

### Conclusions

We used a research through design methodology to craft gamified bodily interactions and explore their potential as antisedentary interventions within online meeting contexts. In collaboration with 11 users, we co-designed and iterated 3 prototypes, which led to the development of the BIG-AOME framework. Using these prototypes, we conducted user studies with 3 groups totaling 15 participants. Empirical findings were gathered to understand user experiences with these prototypes and concretize the framework. The research findings indicate that antisedentary bodily interactions designed for online meetings have the potential to reduce sedentary behaviors while enhancing social connections. Furthermore, the proposed BIG-AOME framework explores the design space for antisedentary physical interactions in the context of online meetings.

## Appendix

Multimedia Appendix 1 Video demonstration.

Multimedia Appendix 2 Gamified bodily interaction evaluation scale.

Multimedia Appendix 3 Semistructured interview guide.

## Abbreviations

BIG-AOME: Bodily Interaction Gamification towards Anti-sedentary Online Meeting Environments
RQ: research question
