# Supplementary material for: Playful Antisedentary Interactions for Online Meeting Scenarios: A Research Through Design Approach
Source: JMIR Serious Games. 2025 Apr 18;13:e62778. doi: 10.2196/62778 (PMC12048789; doi:10.2196/62778)
Supplement: Multimedia Appendix 2 [file games_v13i1e62778_app2.pdf]

Gamified Bodily Interaction Evaluation Scale

The scale is designed to assess user experiences with gamified bodily interactions during online meetings. It measures multiple dimensions, including exertion levels, game duration, narrative importance, physical engagement, attention requirements, and collaborative dynamics within the virtual environment. This scale offers structured insights into user preferences, gameplay effectiveness, and design considerations for anti-sedentary interventions in virtual meeting contexts.

1. How much physical exertion does the game require?
This question evaluates the level of physical activity demanded by the game.
Scale: 1 (Very Low) ～ 5 (Very High)

2. How long should the game ideally last?
This question seeks to understand players' preferences for the duration of the game.
Scale: 1 (5 minutes) ～ 5 (Entire Meeting Duration)

3. How important is the narrative to the game's experience?
This question explores the extent to which the storyline enhances the game's appeal.
Scale: 1 (Focus on Body Movement Only) ～ 5 (Narrative is Fun and Essential)

4. Which areas of the body does the game exercise the most?
This question identifies whether the game focuses on specific body parts or provides a full-body workout.
Scale: 1 (Specific Part Only) ～ 5 (Entire Body)

5. To what extent can players share space, objects, or interactions in the virtual world?
This question assesses the degree of collaborative interaction supported by the game.
Scale: 1 (No Sharing) ～ 5 (Fully Shared Space/Objects)

6. How much attention does the game require to be fully experienced?
This question measures the level of focus needed for optimal enjoyment of the game.
Scale: 1 (Can Multitask) ～ 5 (Full Attention Required)
