# Supplementary material for: Playful Antisedentary Interactions for Online Meeting Scenarios: A Research Through Design Approach
Source: JMIR Serious Games. 2025 Apr 18;13:e62778. doi: 10.2196/62778 (PMC12048789; doi:10.2196/62778)
Supplement: Multimedia Appendix 3 [file games_v13i1e62778_app3.pdf]

Semi-Structured Interview Guide for Evaluating Gamified Bodily Interactions

The following questions were used as a guideline during the semi-structured interviews conducted with participants. These questions aimed to explore their experiences with the gamified bodily interactions and the usability of the prototypes:

General Experience

1. How did you feel about incorporating bodily movements into the games during the meeting?
2. What were your initial impressions of using these games as part of an online meeting?
3. Did the games make the meeting experience more enjoyable or engaging for you? Why or why not?

Physical Interaction

4. Were the physical movements required by the games comfortable and easy to perform?
5. Did you feel any physical strain or fatigue while playing the games? If yes, which game(s) caused this?
6. How do you think the games influenced your physical activity levels during the meeting?

Cognitive and Emotional Engagement

7. To what extent did the games require your focus and attention?
8. Did the games interfere with your ability to concentrate on meeting content? Why or why not?
9. Did you find the games to be mentally refreshing or distracting during the meeting?

Social Interaction

10. How did the games impact your interactions with other participants?
11. Did you feel that the games helped foster a sense of connection or teamwork? Why or why not?

Design Feedback

12. Which game did you enjoy the most and why?
13. Were there any aspects of the games you found confusing or difficult to use?
14. How could these games be improved to better suit online meeting contexts?

Use Case and Application

15. In what types of meetings (formal, informal, team-building) do you think these games would be most appropriate?
16. Would you consider using such games in your own online meetings? Why or why not?
17. What additional features or changes would make these games more appealing for regular use?
